# First identification of ORF virus causing contagious ecthyma in Morocco (MOR20): Genomic, phylogenetic, and sequence variants analyses for vaccine design

**DOI:** 10.1371/journal.pone.0323383

**Published:** 2025-05-12

**Authors:** Zouhair Elkarhat, Ikram Tifrouin, Zahra Bamouh, Khalid Omari Tadlaoui, Mehdi Elharrak

**Affiliations:** 1 Research and Development, MCI Santé Animale, Mohammedia, Morocco; 2 Physiopathology, Molecular Biology and Biotechnology Laboratory, Faculty of Sciences Ain Chock, University Hassan II, Maarif, Casablanca, Morocco; Katholieke Universiteit Leuven Rega Institute for Medical Research, BELGIUM

## Abstract

The ORF virus induces a zoonotic contagious ecthyma disease, affecting small ruminants such as sheep and goats. ORF virus has not been identified in Morocco, and there is no vaccination protocol against contagious ecthyma. In this study, we analyzed the genome sequence of a new strain isolated in Morocco (MOR20) from a flock of sheep showing suspicious signs of Sheepox virus infection. ORFV MOR20 strain was isolated after 2 initial blind passages on Heart cells. The cytopathic effect was characterized by aggregation, swelling and detachment of cells, appearing 4 days after infection. The virus was harvested on day 6 pi with a titer of 10^7.2^ TCID/ml. ORFV MOR20 was sequenced using the Illumina NovaSeq 6000 platform. After employing several bioinformatics tools, we identified that ORFV MOR20 shares 98.59% similarity with the TVL strain virus, which is used in a commercial live vaccine. Additionally, We aligned 33 ORFV genomic sequences with MOR20 sequences and visualized the pairwise comparisons using a Heat Map. ORFV was classified into two genetic groups: those isolated from sheep and those from goats. This was confirmed by a phylogenetic tree. Furthermore, we analyzed genetic variants identified in the MOR20 strain in comparison with ORFV TVL strain and found 636 sequence variants. Some genes, such as ORFV086, ORFV112, and ORFV132, have a particularly high number of sequence variants. All in all, ORFV MOR20 isolate represents a promising candidate for further studies aimed at developing a standardized vaccine against contagious ecthyma.

## Introduction

The ORF virus (ORFV) belongs to the Poxviridae family, genus Parapoxvirus [1]. It is the agent of Contagious Ecthyma (CE), a zoonotic disease, also known as contagious pustular dermatitis (CPD), infectious labial dermatitis, scabby mouth, sore mouth. According to the International Committee on Taxonomy of Viruses, the Parapoxvirus genus includes four other species: Bovine papular stomatitis virus (BPSV), pseudocowpox virus (PCPV), red deer pox virus (RDPV) and gray pox virus [2].

Contagious ecthyma mainly affects sheep, goats and various other small ruminants [3–5]. It manifests as proliferative skin lesions around the oral region, presenting as erythematous macules, papules, vesicles, pustules and crusts [3–6]. This disease is non-systemic and mainly affects the skin, with a worldwide distribution [7]. Although sheep and goats are the main hosts, other animals such as camels, deer, reindeer, musk oxen and Japanese serow are also susceptible to infection [8]. The virus is highly contagious and remains infectious for extended periods. It has been found to survive for up to 17 years in environments with dry climates and remain viable in wools, animal excreta and contaminated material for significant long periods [9]. Clinical manifestations often involve proliferative lesions on the mouth and muzzle, resolving typically within 1–2 months [10]. These lesions may interfere with suckling in lambs and feeding in neonates due to udder involvement [11]. ORFV presents a potential life-threatening risk for neonates as it disrupts suckling from the infected udder and predisposes the animals to secondary bacterial or fungal infections, potentially leading to a mortality rate of up to 15% [12]. Additionally, foot lesions can induce transient lameness, contributing to poor health and weight loss. Although lesions progress through clinical stages, they generally resolve within 2–3 weeks without significant proliferation [11].

ORFV is a large, ovoid, enveloped, double-stranded positive-sense DNA virus with a linear genome of 130–140 kb and measuring approximately 260 nm × 160 nm. The DNA has a high G + C content, reaching up to 63% [11,13,14]. This G + C content is remarkable among poxviruses, giving ORFV a distinctive genomic composition [13,15]. Furthermore, the ORFV genome has inverted terminal repeats at the ends, which suggests a common evolutionary origin with other poxviruses, notably vaccinia virus [16].

The genome includes 132 open reading frames (ORFs), 88 of which are conserved among chordopoxviruses [17–19]. This genomic structure organizes ORFV genes into distinct regions, with genes essential for replication, transcription, and virion assembly concentrated in the central region, while genes associated with virulence, immune modulation, and to pathogenesis are often located near the variable endings. These structural and dimensional characteristics of the ORFV genome define its biological properties and influence its interactions with hosts and its pathogenic potential. Virulence genes are mainly located in terminal regions, rich in genes associated with host specificity and pathogenesis [9,17].

The immunomodulatory proteins (IMPs) of ORFV play a crucial role in its ability to cause disease, some of which have been identified as key factors in regulating host immune responses [2,13,20]. These IMPs, such as the chemokine binding protein (CBP), soluble protein inhibitor of granulocyte-monocyte colony-stimulating factor and interleukin-2 (GIF), and the orthologue of ovine interleukin-10 (vIL-10), are essential for ORFV virulence, as demonstrated by gene deletion studies [21–28].

ORFV has at least two types of infectious particles: the mature virion, characterized by its outer membrane derived from the endoplasmic reticulum, and the extracellular virion, which comes from the enveloped virion form. This extracellular virion is enveloped by two additional membranes, derived from the trans-Golgi apparatus, after losing its outermost membrane during egress [29]. Through the production of extracellular virion particles, the ORFV can spread efficiently from one cell to another. ORFV replication primarily occurs in the host cell cytoplasm, requiring its own transcription and replication machinery. Genes essential for these processes are concentrated in the central genome region. Several ORFV genes have been characterized for their function, including envelope proteins with lipase activity (B2L gene) and immunogenicity (F1L gene), as well as genes involved in interferon resistance (VIR gene), an anti- apoptotic (ORF125 gene) and type II envelope glycoproteins (ORF109 gene) involved in the assembly and invasion of viral particles. GIF is the only known viral protein capable of inhibiting host GM-CSF and IL-2 activity, highlighting the multifaceted strategies of ORFV in immune modulation and pathogenesis [18,30–34].

Orf virus has not previously been identified in North Africa except Egypt [35] and that this is the first report of Orf virus in Morocco, and the first genome sequence in North Africa. The aim of this study is to conduct a genomic analysis on the recent ORF isolate, compare its sequence with the genomes deposited at NCBI database, construct a phylogenetic tree and identify the sequence variants relative to the reference sequence.

## Materials and methods

### Virus isolation

In 2020, the ORF virus was suspected in a flock of sheep in the Gharb Chrada Beni Hssen region of northwestern Morocco, displaying signs suspiciously similar to Sheeppox virus (SPPV) infection. The skin scabs were minced, ground, and suspended in phosphate-buffered saline (PBS) with 5% penicillin and streptomycin. The homogenized suspension was then centrifuged at 5000 rpm for 30 min and the supernatant was filtered through a 0.45 µm membrane. The filtered supernatant was tested on primary lamb hearth cells. Cells were cultured and maintained in a sterile 75 cm2 flask using Dulbecco’s Modified Eagle Medium (DMEM) cell culture medium supplemented with 10% fetal bovine serum (FBS). Cells were inoculated by 500 μl samples into 25 cm2 flasks. The control cells were inoculated with 500 μl of PBS. This study was carried out in strict accordance with international guidelines for care and handling of experimental animals, chapter 7.8 of the Terrestrial Animal Health Code and Directive 2010/63/UE of the European Commission. The protocol was approved by the by the Internal Ethics Committee of MCI Santé Animale (Protocol number PRT-RD006).

### PCR identification

Viral DNA was extracted from harvested cells on day 6 pi using ISOLATE II Genomic DNA Kit (Bioline, London, UK) according to the manufacturer’s instructions. Virus identity was analysed by qPCR using specific primers for ORF virus [36]. Briefly, qPCR was conducted using the TaqMan Universal PCR Master Mix Kit according to the manufacturer’s instructions (Applied Biosystems, Foster City, CA, 160 USA). qPCR was conducted in 96-well optical reaction plates, with each well containing the following components: 12.5 µ L of 2 × TaqMan Universal PCR Master Mix, Real-time PCR buffer, 1 µ L of each primer (Forward: 5′- CAGCAGAGCCGCGTGAA -3′ and Reverse: 5′-CATGAACCGCTACAACACCTTCT-3′) at a concentration of 10 µ M, 0.5 µ L of probe (FAM- CACCTTCGGCTCCAC -MGB) at a concentration of 10 µ M, 5 µ L of template (DNA), and 5 µ L of nuclease-free water. The qPCR assay was run in ABI7500 (Applied 165 Biosystems) with the following cycling conditions: 95°C for 10 min, followed by 40 cycles of PCR at 95°C for 15 s and 58°C for 1 min.

### Sequencing

The ORF virus genome was sequenced via a special service for DNA virus sequencing (INVIEW virus sequencing, Eurofins Genomics Europe Sequencing GmbH Ebersberg, Germany) using the llumina NovaSeq 6,000 platform (2 × 150 sequence mode). The DNA extract was qualified and quantified by agarose gel electrophoresis and analysis by NanoDrop/Qubit Fluorometer. In parallel, DIN value estimation was performed using the Agilent 4200 Tape Station to assess DNA integrity. For the preparation of the sequencing libraries, the Illumina TruSeq Nano DNA kit was used, opting for a fragment size adapted to the virus ORF sequence. The libraries thus prepared were subjected to further quality control using the Agilent 4200 Tape Station to ensure their quality and integrity. Finally, the libraries were sequenced on the NovaSeq 6000 platform with 2x150 bp chemistry and the raw data were retrieved as compressed FASTQ files.

The quality of raw sequencing data was assessed by checking base calling accuracy and removing low-quality bases. This was achieved by performing adapter trimming, quality filtering, and per-read quality pruning. Then, the data were processed using fastp software to remove low quality bases (below Phred 20 quality) using the sliding window approach [37]. The remaining adapters were removed, and reads shorter than 30 bp were discarded. After this process, high-quality sequencing reads were retained for each sample. Q30 reads, representing a base calling accuracy of 99.9%, were used as a quality indicator, with the goal of obtaining a high percentage of Q30 reads in our sample. Metrics such as GC content were used to assess overall sequencing and sample quality.

### Genome mapping

Preprocessed data were mapped to the reference sequence using BWA, run through Sentieon framework [38]. The reads were then classified into different categories such as mapped, unique, non-unique, singletons and cross-contig, depending on their alignment to the reference. Unwanted reads, such as non-unique reads, singletons, and cross-contig reads, were removed from the dataset. Unique reads were selected for further analyses. Additionally, reads were deduplicated to remove any artificial coverage introduced by PCR amplification during library preparation and/or sequencing using sambamba [39]. Base quality recalibration was performed to improve the accuracy of read quality scores, using GATK tools that consider various sequencing characteristics. Finally, detailed alignment metrics were extracted to assess sequencing and sample quality. This robust methodology ensures the accuracy and reproducibility of the results obtained from the sequencing data, providing a solid basis for subsequent analyzes in this study.

### Sequence variant detection and annotation

The SNPs (single nucleotide polymorphisms) and InDels (insertions/deletions) calling was performed using Sentieon’s HaplotypeCaller. The detected sequence variants were annotated based on their gene context using snpEff [40]. After annotation, Customized filters were applied to the variants to filter out false positive variants using the Variant Filtration module of GATK [41,42]. Filters include ReadPosFilter for read position, MQRankSumLow for sum of mapping quality ranks, LowCovFilter for coverage depth, QDFilter for quality by read depth, MQFilter for average mapping quality, FSFilter for bias strand, and HaplotypeFilter for haplotype consistency. These filters were specifically designed to remove lower quality variants while retaining high quality variants, thereby contributing to the reliability and accuracy of the variant calling results in this study. Finally, variants with 100% of frequency were selected for further analyses.

The consensus sequence was extracted from the BAM files obtained after mapping the FASTQ files to the reference sequence using the Extract Consensus Sequence tool of the CLC genomic workbench software. This method allowed us to generate a FASTA file containing the consensus sequence representative of the regions aligned to the reference sequence, thereby providing a consolidated representation of the sequencing data for subsequent analysis.

### Blast genome analysis

The consensus sequence of the isolated ORFV was compared to ORFV genome sequences deposited in the NCBI database. We aligned 33 ORFV genomic sequences with our ORFV genomic sequence using the Whole Genome Alignment tool available in the CLC Genomic Workbench software. The tool was configured with a minimum initial seed length of 15 nucleotides and a minimum alignment block length of 100 nucleotides. Subsequently, we further analyzed the dataset using the Average Nucleotide Identity Comparison tool available in the software. This tool allows quantitative assessment of genomic similarity using the whole genome alignment as input. For each pair of genomes, the tool identifies aligned regions and calculates two key metrics: alignment percentage and average nucleotide identity. Finally, we proceeded to visualize the pairwise genomic comparisons using the Heat Map from Comparison tool. This method constructs a heat map based on the pairwise comparison results, such as those derived from the Average Nucleotide Identity Comparison tool. To ensure the accuracy and reliability of the heatmap, we configured the tool with specific parameters: a minimum similarity fraction of 0.8 and a minimum length fraction of 0.8. These thresholds were chosen to strike a balance between sensitivity and specificity in detecting meaningful genomic relationships. The resulting heat map provides a comprehensive overview of the genomic relationships between ORF virus strains.

### BLAST gene analysis

The consensus sequence of the ORFV112 gene was extracted from the whole genome sequence and compared to the ORF112 gene sequences of all 33 entries in the NCBI database using the NCBI BLAST nucleotide tool. Additionally, we compared the ORF112 genes of the 33 strains with each other to identify the most similar strains.

### Phylogenetic analysis

The FASTA sequences of the 34 ORFV virus genomes were submitted to the BuscoPhylo webserver for Busco-based phylogenomic analysis [43]. The multiple sequence alignment generated is used to infer the maximum likelihood (ML) tree using IQ-TREE. By default, IQ-TREE determines the best-ft substitution model using ModelFinder. The tree was then modified by the iTOL tool.

## Results

### Virus isolation

The virus was isolated after 2–3 initial blind passages on Hearth diploid cells. The cytopathic effect was characterized by aggregation, swelling and detachment of cells, appearing 4 days after infection (pi). The virus was harvested on the day 6 pi with a titer of 10^7.2^ TCID/ml. Real time PCR using specific primers for ORFV confirm the identity of the isolate named ORF MOR20 with a Ct value of 19.46.

### Assessment of raw data quality parameters

Sequencing data revealed high quality metrics for the sample ORFV. It generated a total of 14.68 million raw reads, which were efficiently cleaned and filtered to produce 14.5 million high-quality reads (Table 1). These high-quality reads demonstrated a high percentage of high-quality bases, reaching 93.5% with a Phred quality score of 30 or higher. Additionally, the sample had a GC content of 49.2% among its high-quality sequences, with an average read length of 149 bp. The overall quality of high-quality reads was remarkable, accounting for 98.8% of all reads generated.

**Table 1:** Sequence Quality Metrics overview.

Sample	Total Raw Reads	Total HQ Reads	HQ Bases (Q30)	GC Content	Mean Read Length (bp)	HQ Reads %
ORF MOR20	14.68 M	14.5 M	93,5%	49,2%	149	98,8%

### Mapping efficiency and coverage analysis

In our analysis, we examined the mapping statistics of the ORF MOR20 sample to evaluate the effectiveness of our approach. Following rigorous filtering processes, a total of 14.48 million high-quality reads were retained for analysis (Table 2). Of these, 3.24 million reads, or 22.4% of the total, were successfully mapped to the reference genome. Impressively, all mapped reads were unique, demonstrating the accuracy of the alignment, with 83.1% of them lacking PCR duplicates after deduplication. The average coverage of the reference sequence was an impressive 3,517.32x, demonstrating robust sequencing depth. Notably, after removing duplicates, the average coverage remained substantial at 2947.46x.

**Table 2:** Mapping metrics of ORF MOR20.

Sample	Total HQ Reads	Mapped Reads	Unique Reads	Deduplicated Reads	Mean Coverage	Mean Coverage (w/o duplicates)
ORF MOR20	14.48M	3.24M (22.4%)	3.24M (22.4%)	2.69M (83.1%)	3517.32x	2947.46x

### Coverage depth

Assessment of the depth of coverage is crucial to understand the reliability and completeness of genomic sequencing. At 2x coverage, 93.2% of the reference sequence was covered, with high coverage persisting even at stringent thresholds such as 120x, where 92.4% of the reference remained covered (Table 3). Post-duplicate removal, the mean coverage remained substantial at 2947.46x, indicating robust sequencing depth even after eliminating PCR duplicates. These findings underscore the thoroughness and quality of the sequencing data obtained, laying a solid groundwork for comprehensive genomic analyses.

**Table 3:** Depth of coverage summary.

Sample (ORF MOR20)	Mean Coverage	2x	5x	10x	20x	30x	60x	90x	120x
before duplicate removal	3517,32	93,2	92,8	92,7	92,6	92,6	92,5	92,4	92,4
After duplicate removal	2947,46	93	92,8	92,7	92,6	92,5	92,5	92,4	92,4

### Blast with genome sequences

The BLAST of the consensus sequence of the Moroccan strain with 33 genomes sequences of ORF virus isolated from different countries revealed that ORFV MOR20 shares highest sequence similarities with ORFV viruses isolated from sheep, particularly the TVL strain from USA, S6, S10 and S27 strains from Italy, NAV strain from Spain and NZ2 strain from New Zealand (Table 4). In addition, the sequence similarity between ORFV MOR20 and B029 strain isolated from Human in Germany is around 98.39% with a 92% of query cover. The isolate showed the lowest similarity (97.26%) with the virus isolated from goats in India and China. The consensus sequence of ORF MOR20 strain was submitted to GenBank with accession number PQ685033.

**Table 4:** Comparison of ORFV MOR20 strain with ORFV isolated from, sheep, goat and human using NCBI BLAST.

Description	Country	Collection date	Host	Query Cover	Percentage of Identity	Accession
TVL strain	USA	2019	Sheep	92%	98.59%	MN454854.1
S6 strain	Italy	2020	Sheep	91%	98.51%	ON691519.1
S10 strain	Italy	2020	Sheep	90%	98.49%	ON691520.1
S27 strain	Italy	2019	Sheep	91%	98.46%	ON691524.1
NAV strain	Spain	2018	Sheep	92%	98.44%	ON805832.1
NZ2 strain	New Zealand	Unknown <1982	Sheep	92%	98.43%	DQ184476.1
OV-IA82 strain	USA	1982	Sheep	92%	98.41%	AY386263.1
S21 strain	Italy	2017	Sheep	90%	98.40%	ON691523.1
B029 strain	Germany	1996	Human	92%	98.39%	KF837136.1
S19 strain	Italy	2021	Sheep	91%	98.39%	ON691522.1
ARA strain	Spain	2018	Sheep	92%	98.39%	ON805833.1
CHB strain	Argentina	2018	Sheep	92%	98.38%	ON805830.1
HRE strain	Argentina	2018	Sheep	92%	98.35%	ON805831.1
SY17 strain	China	2016	Sheep	92%	98.32%	MG712417.1
OV-HN3/12 strain	China	2012	Sheep	92%	98.29%	KY053526.1
NA1/11 strain	China	2011	Sheep	92%	98.28%	KF234407.1
S30 strain	Italy	2019	Goat	91%	98.02%	ON691525.1
S15 strain	Italy	2020	Goat	91%	97.97%	ON691521.1
GZ18 strain	China	2018	Goat	92%	97.93%	MN648218.1
CL18 strain	China	2018	Sheep	92%	97.89%	MN648219.1
NP strain	China	2011	Goat	90%	97.79%	KP010355.1
OV-SA00 strain	USA	2004	Goat	92%	97.78%	AY386264.1
nm-W strain	China	2020	Goat	88%	97.64%	OP151442.1
NA17 strain	China	2016	Goat	92%	97.60%	MG674916.2
YX strain	China	2012	Goat	92%	97.60%	KP010353.1
SJ1 strain	China	2012	Goat	91%	97.59%	KP010356.1
D1701 strain	Germany	Unknown < 2010	Sheep	91%	97.58%	HM133903.1
UPM/HSN-20 strain	Malaysia	2022	Goat	91%	97.58%	MW537048.1
GO strain	China	2012	Goat	91%	97.56%	KP010354.1
MP strain	India	2017	Goat	91%	97.55%	MT332357.1
Mukteswar passage 9 strain	India	2005	Goat	92%	97.46%	ON380499.1
Mukteswar vaccine P50 strain	India	2005	Goat	92%	97.45%	ON380500.1

### Multi-alignment of the genome sequences

**Fig 1:**
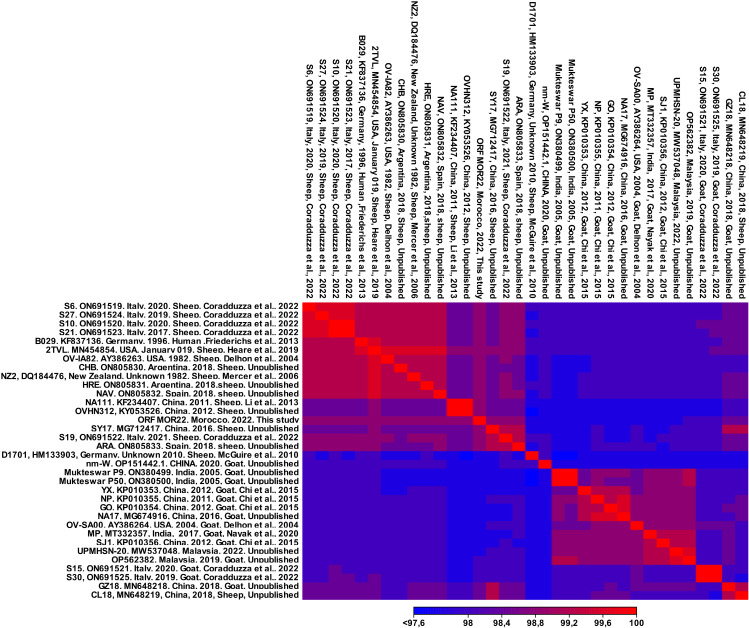
Heatmap of whole-genome sequence pairwise comparisons between ORFV strains isolated from sheep and goats.

Pairwise genomic comparisons analysis reveals two categories of ORF virus genomic sequences: those isolated from sheep and those from goats. The analysis demonstrates that the 14 genomes of the ORF virus isolated from goats exhibit significant sequence similarity among themselves and are less similar to viruses isolated from sheep. Similarly, the 17 ORF viruses isolated from sheep show high similarity to each other but are less similar to viruses isolated from goats Fig 1.

### ORF112 gene analysis

**Fig 2:**
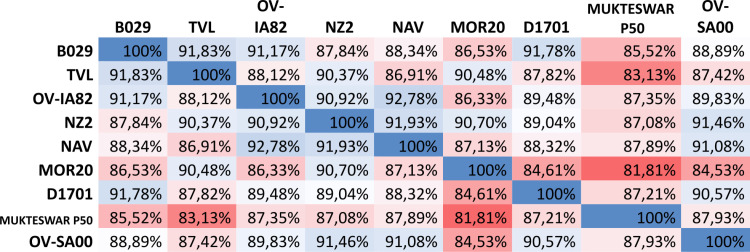
Heatmap of ORFV112 gene similarities with ORFV strains isolated from sheep and goats.

ORFV112 gene of TVL strain shows high similarities (between 86.91% and 91.83%) with most other strains, except with MUKTESWAR P50 (83.13%) (Fig 2). This indicates that TVL is overall closer to all other strains. However, ORFV112 gene of MOR20 strain has lower similarities (between 81.81% and 90.70%) with the other strains, showing a more marked divergence, in particular with D1701 (84.61%), MUKTESWAR P50 (81.81%), and OV-SA00 (84.53%). However, MOR20 shows a notable proximity to NZ2 (90.70%) and TVL (90.48%) (Fig 2).

### Phylogenetic analysis

**Fig 3:**
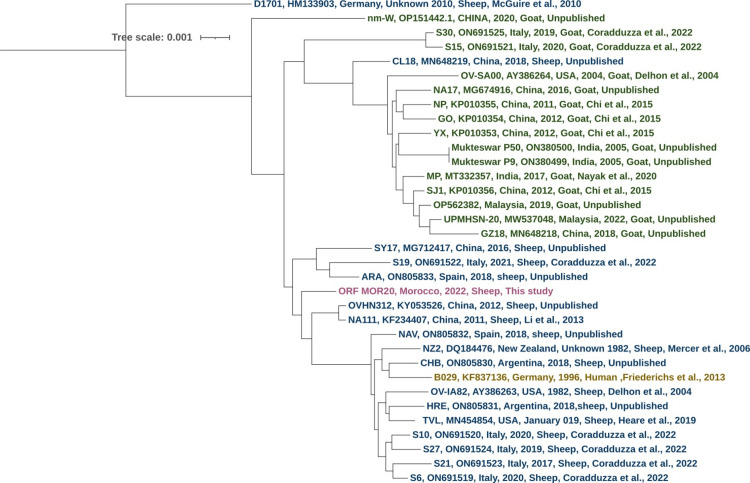
Phylogenetic tree of whole-genome sequences comparisons between ORFV strains isolated from sheep and goats.

Phylogenetic tree analysis of all ORFV genome sequences confirms the distinction of sequences into two groups; isolates from goats and isolates from sheep. Morocco isolate was categorized with strains from sheep within the same major clade (Fig 3).

### Genetic variation

The comparative analysis of ORFV MOR20 sequence with the ORFV TVL strain revealed the presence of 636 sequence variants (Table 5). Among them are 322 synonymous variants, 167 missense variants, 14 frame shift variants, 5 disruptive inframe deletions, 4 disruptive inframe insertions, 2 conservative in-frame deletions, and 5 other types of variants. Almost all genes have variants. ORFV086 gene contains the highest number of variants [21], followed by ORFV132 [16] and ORFV112 (Table 6). Table 7 presents the missense variants present in all genes.

**Table 5:** Distribution of sequence variant types in the ORFV MOR20 genome.

Variants type	Number	Frequency
synonymous_variant	322	50,63
missense_variant	167	26,26
Intergenic variants	120	18,87
Frameshift variant	14	2,20
Disruptive inframe deletion	5	0,79
Disruptive inframe insertion	4	0,63
Conservative inframe deletion	2	0,31
Frameshift variant & splice region variant	1	0,16
Splice region variant & stop retained variant	1	0,16
Stop lost & splice region variant	1	0,16
Conservative inframe insertion	1	0,16
Frameshift variant & stop gained	1	0,16
Total	636	

**Table 6:** Distribution of sequence variants in ORFV genes.

GENE	PROTEIN	SEQUENCE VARIANTS
***ORFV086***	major core protein 4a precursor protein	21
***ORFV132***	vascular endothelial growth factor	16
***01orf_00022***	DNA binding protein	15
***ORFV112***	chemokine-binding protein	15
***ORFV113***	hypothetical protein	15
***ORFV008***	ankyrin/F-box protein	13
***ORFV016***	hypothetical protein	13
***ORFV024***	NF-kappaB pathway inhibitor	12
***ORFV128***	ankyrin/F-box protein	12
***VETFL***	early transcription factor	10
***G9R***	myristoylated G9 protein	9
***NPH2***	NPH-II RNA helicase	9
***G7L***	G7 assembly protein	5
***POL***	DNA polymerase	5
***PAPL***	poly(A) polymerase catalytic subunit	4
***VITF3L***	the large subunit of intermediate transcription factor 3	4
***E6R***	E6 protein	3
***VPK2***	serine/threonine-protein kinase 2	3
***NPH1***	nucleoside triphosphatase I	2
***PAPS***	Cap-specific mRNA	2
***UNG***	uracil-DNA glycosylase	2
***VETFS***	70 kDa early transcription factor subunit,	2
***VLTF1***	late transcription factor 1	2
***L5R***	L5 protein	1
***TOP1***	DNA topoisomerase 1B	1
***VITF3S***	small subunit of intermediate transcription factor 3	1
***VLTF2***	late viral gene transcription factor 2	1

**Table 7:** Missense variants identified in the ORFV MOR20 genome.

LOCUS TAG	AMINO ACID CHANGE	LOCUS TAG	AMINO ACID CHANGE	LOCUS TAG	AMINO ACID CHANGE	LOCUS TAG	AMINO ACID CHANGE
**01orf_00001**	R515H; Q445P; M245L; G228E; C222R	**01orf_00027**	D278E; D132E; S23P	**01orf_00068**	V184I	**01orf_00104**	N48D; D135N
**01orf_00003**	D96N; D45N	**01orf_00031**	V85A; A388V	**01orf_00060**	P11L	**01orf_00105**	F58L; T60A; G226R; F251V; K254R; M258I; K262Q; D284E
**01orf_00004**	V185I; N79D	**01orf_00032**	D405N	**01orf_00071**	E80G; Q57R	**01orf_00106**	V7I; E96G; T158A; V161M; R164S; N166D; N191T
**01orf_00005**	R76K	**01orf_00035**	G308R; V343A	**01orf_00075**	V288I; S150N; P95A	**01orf_00107**	S303G
**01orf_00008**	K85E; R9K; K6R	**01orf_00037**	T148I	**01orf_00076**	A563T	**01orf_00110**	M114VL145PH155Y
**01orf_00009**	V519M; M53L	**01orf_00038**	A385V	**01orf_00079**	T470M; A445T; A371T; C143Y; T101A; V87I	**01orf_00073**	R40C; A20V
**01orf_00010**	N226S; S184P; G99S; E35G; E35Q; M27V	**01orf_00040**	G138S; Q159R; A177T; V302A	**01orf_00081**	T185A; V146I; C120Y	**01orf_00112**	D85E; Y89H; E99D; G137S; G177S; G243D; G250E
**01orf_00011**	M17I; S60P	**01orf_00043**	A96V	**01orf_00082**	P76A	**01orf_00113**	M206K; T244I; I304V
**01orf_00013**	R589K; A450V; A432T; T160A	**01orf_00045**	V8A	**01orf_00084**	F22L	**01orf_00114**	G175S; A234V; H334R; D464G
**01orf_00014**	I170S; S124P; D17G	**01orf_00047**	V134I	**01orf_00086**	G235D	**01orf_00115**	D38G; Q426R; G435R; G435E
**01orf_00016**	A91T	**01orf_00050**	L855V; E1108G	**01orf_00087**	V44A	**01orf_00116**	G161S
**01orf_00018**	H24Q; R37C; R43C; P64S; G120R; G124S	**01orf_00053**	A152T	**01orf_00091**	D226N	**01orf_00117**	T132A; A138V
**01orf_00022**	H310R; R249K; S196N; N165S; H157R; Q144R; M44T	**01orf_00054**	G549D; G549S; E35D	**01orf_00092**	A1144T	**01orf_00119**	E35A; R38C; V39A; D40E; F248L; T309A
**01orf_00023**	A590V; N387S	**01orf_00055**	S39N; T69P; A100T; I164L; A218T	**01orf_00093**	A36P	**01orf_00120**	D12N; V376A; V366A; A414T
**01orf_00024**	R50Q; H35Y	**01orf_00057**	A138V	**01orf_00094**	L186F	**01orf_00122**	K94R
**01orf_00025**	S316P	**01orf_00059**	V32A	**01orf_00099**	D152E	**01orf_00123**	N23S; S28P; K29E; K32E; E35K; V43G; V84A

## Discussion

Contagious ecthyma is a highly contagious zoonotic disease with global distribution, that primarily affects sheep, goats, and other small ruminants [44]. It is caused by the ORF virus of the parapoxvirus family [45]. In North Africa, the contagious ecthyma disease has only been reported in Egypt [46], and no isolated virus has been sequenced or molecularly studied. This study reports molecular characterization of the first Moroccan and North African isolate of the ORF virus. The MOR20 strain was isolated from an outbreak of SPPV in a sheep herd on heart diploid cells, after 3 blind passages. We observed a cytopathic effect with high viral titer, demonstrating the viability and infectious potential of the isolated virus on cells. The identity of the ORFV virus was initially confirmed by specific real-time PCR.

A comprehensive genomic analysis of the virus was conducted to molecularly characterize the strain and compare it with other worldwide isolates. The sequencing of the MOR20 ORF strain generated a remarkable 98.8% of high-quality reads. The high-quality reads were aligned to TVL strain collected from domestic sheep in pasture in West Texas, USA [2,4]. Indeed, 22.4% of the reads successfully mapped to the reference genome with a robust coverage depth, affirming the efficacy of the conducted protocol. Thus, we obtained that our genome shares the highest similarity with ORFV viruses isolated from sheep, particularly with TVL and the S6 strain [2,4]. Moreover, our sequence exhibits the lowest similarity with the virus isolated from goats in India, Mukteswar vaccine P50 strain, which underwent 50 passages on cells.

Regarding the distribution patterns of ORF virus isolates, the Heat Map and the phylogenetic analyses revealed that ORF MOR20 strain showed high similarity with strains isolated from sheep, while displaying reduced similarity with strains from goats. According to the previous studies, we also observed a genetic distinction between ORFV isolated from sheep and goats indicating a shared evolutionary lineage and potential host-specific adaptations within the ORFV population [2]. Moreover, we also identified a close link between the ORFV strain isolated from human, B029 strain, and those identified in sheep. This suggests that circulation of the virus between sheep and humans may be more frequent due to the greater number of sheep farms compared to goats. In addition, We identified that strain CL18, isolated from a sheep, is present in the clade of viruses isolated from goats. This strain could represent a genetically distinct variant of ORFV capable of infecting both sheep and goats, or it could show a preference for one host while retaining the ability to infect immunocompromised individuals of the other species. Such cross-species infections are more likely to occur and spread in regions where sheep and goats are raised in close contact. In China, these two species are commonly raised together, a traditional practice that likely explains the comparable numbers of sheep and goats currently observed in the country [47,48].

Analysis of genetic variants in MOR20 genome sequence revealed that almost all genes presented variants. The gene that contains the most sequence variants is the ORFV086 gene that codes for a structural protein expressed in the mature intracellular core of the virus, named major precursor protein core protein 4a. This protein shows strong similarity to the vaccinia virus core precursor protein, P4a, and other poxvirus homologs [49–51], its high expression in vaccinia virus is essential for the formation of mature infectious progeny of the virus [50,51]. Wang et al. identified that the ORFV086 protein was located in the “virus factory” and could be observed as early as 12 hours pi., indicating that ORFV086 is a late gene product [52]. Indeed, this protein is essential for viral particle assembly, morphogenesis, and maturation, playing a crucial role in the formation of infectious viral progeny.

We also found several sequence variants in the ORFV132 gene, which codes for vascular endothelial growth factor (VEGF). ORFV132 is an immunomodulatory gene located in the highly variable terminal regions at the right end of the conserved region [29,53–57]. The VEGF protein has putative virulence functions. It promotes the continued proliferation of epithelial cells, which facilitates the creation of binding sites for ORFV replication [54,56,57]. It also protects the virus from the effects of the immune response and neutralizes the effects of host antiviral apoptosis [57–59]. Recent studies have shown that inactivation of the VEGF protein attenuates the virus and reduces the severity of disease in host cells [53,57,58].

Another gene, ORFV112, which codes for a protein also having putative virulence functions, contains a high number of chemokine-binding (CBP) sequence variants. In this study, we observed that, although, MOR20 and TVL are relatively similar to each other, the degree of similarity of the ORFV112 gene to other strains varies significantly. ORFV112 gene of TVL strain is overall more similar to other strains, while ORFV112 gene of MOR20 strain shows more marked divergence with multiple strains indicating greater variability or more pronounced evolutionary differences. This protein is the first virulence protein synthesized by the virus after successful invasion of the host cell [60,61]. It interacts with IFN-α, IL-8, and IFN-γ, which allows temporary replication of the organism’s viral antigen in infected cells [62,63]. It also inhibits the recruitment and migration of dendritic cells and other immune cells to peripheral lymph nodes to inhibit an adaptive immune response [63,64]. Furthermore, Martins et al. demonstrated that individual deletion of immunomodulatory genes from the ORFV genome, such as ORFV112, ORFV117, and ORFV127, resulted in a slight reduction in virulence in vivo, as evidenced by a reduction in clinical disease duration and shedding of the virus [59]. The presence of sequence variants in genes encoding for proteins involved in key processes such as viral replication can have an impact on the transmission and virulence of the strain. However, the uniqueness of these mutations and their potential functional impact on viral virulence remain to be fully investigated. Some variations may be located in regions of functional importance, such as conserved domains or binding sites, but further in silico and experimental studies are needed to confirm these hypotheses. These analyses will be considered in future work to deepen our understanding of the functional significance of these variations.

It is noteworthy that we conducted an experimental infection study with MOR20 strain (Data submitted for publication). We found that lambs inoculated with MOR20 strain via skin scarification showed moderate clinical signs such as hyperthermia and local lesions, without observing any signs of severe disease or complications. These findings suggest that the MOR20 strain, which has the highest similarity to the attenuated strain of ORFV used in commercial live vaccine (USDA product serial number 1821.51), presents a limited pathogenicity profile in lambs and may be considered non-pathogenic under the experimental conditions employed. This result is very important given that the absence of a vaccination program in Morocco and the presence of the pathogenic sheep pox virus could mask ORF symptoms and allow the strain to circulate unnoticed. This finding indicate also that the MOR20 strain could be used in the development of a live vaccine, which offer a rapid and economical solution to prevent the spread of the virus.

## Conclusion

This study report genomic analysis of a new ORFV strain isolated in Morocco from sheep coinfected by sheep pox virus. The strain shows great similarities to all viruses isolated from sheep compared to those from goats. Genomic analyzes reveal that ORFV could be genetically classified into two classes: ORFV isolated from sheep and others from goats. The new isolate is highly similar to a vaccine strain from genebank.
